# P-2202. Viral Prevalence, Demographic Characteristics, and Clinical Manifestations among Outpatients with Acute Respiratory Infection during Five Influenza Seasons

**DOI:** 10.1093/ofid/ofaf695.2365

**Published:** 2026-01-11

**Authors:** Mary Patricia Nowalk, John V Williams, Monika Johnson, Helen D’Agostino, Gabriella Alicea, Michael Susick, Lora Pless, Richard K Zimmerman, G K Balasubramani

**Affiliations:** University of Pittsburgh, Pittsburgh, PA; University of Wisconsin Madison, Madison, Wisconsin; University of Pittsburgh, Pittsburgh, PA; University of Pittsburgh, Pittsburgh, PA; University of Pittsburgh, Pittsburgh, PA; Universtiy of Pittsburgh, Pittsburgh, PA; University of Pittsburgh, Pittsburgh, PA; University of Pittsburgh, Pittsburgh, PA; University of Pittsburgh, Pittsburgh, PA

## Abstract

**Background:**

Numerous studies, primarily among hospitalized patients, have been undertaken to describe the epidemiology and burden of human metapneumovirus (hMPV). We analyzed stored specimens from an influenza vaccine effectiveness study from the 2016-20 and 2021-22 active influenza seasons to determine the prevalence of several respiratory virus infections including hMPV among outpatients seeking care for an acute respiratory illness (ARI).

**Figure:**
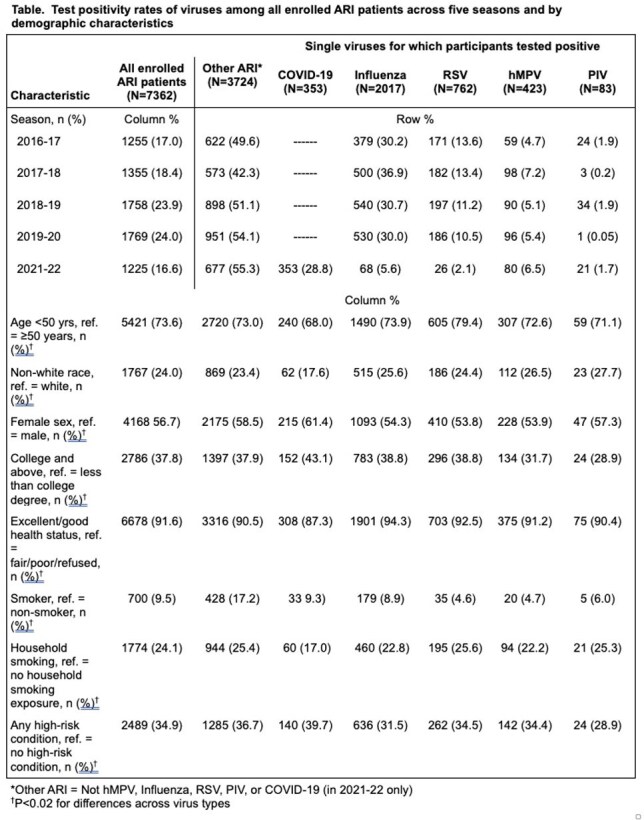

**Methods:**

Consented enrollees were individuals ≥6 months of age presenting with cough of ≤ 7 days’ duration who provided nasal and pharyngeal ( > 24 months only) swabs, access to EMR data and completed enrollment and follow-up surveys. Samples were tested by singleplex reverse transcription polymerase chain reaction (RT-PCR) assays for presence of influenza virus, hMPV, RSV, PIV and SARS-CoV-2 (2021-22 only). Individuals with co-infections were excluded from the study sample. Prevalence of infection was calculated using the percentage of each targeted virus among all ARI tests in a given time period.

**Results:**

After excluding 68 co-infections, 7,362 enrollees were grouped into those with influenza (n=2,017), RSV (n=762), hMPV (n=423), PIV (n=83), SARS-CoV-2 (n=353) and other ARI (who tested negative for any of these viruses (n=3,724). The percentage of total ARI patients each season (exclusive of 2021-22 when SARS-CoV-2 predominated) who tested positive was: 30.0%-36.9% ˗ influenza, 10.5%-13.6% - RSV; 4.7%-7.2% - hMPV; and 0.05%-1.9% - PIV. Compared with patients with influenza, those with hMPV infections were younger, less often were smokers, less often had fever or sore throat, but more often had congestion, shortness of breath (SOB) and follow-up medical visits. Compared with patients with RSV, hMPV patients were older, had higher BMI, and less often had congestion and SOB. Compared with patients with other ARI, hMPV patients were younger, less often reported smoking or sore throat and more often reported fever and follow-up medical visits.

**Conclusion:**

hMPV represented 4.7-7.2% of ARI cases presenting for outpatient care during the 2016-20 and 2021-22 active influenza seasons. Individuals with hMPV infections had different demographic characteristics and presented with distinct symptoms from those with influenza, RSV or other ARI.

**Disclosures:**

Mary Patricia Nowalk, PhD, Abbott labs: Stocks/Bonds (Public Company)|AstraZeneca - Icosavax: Grant/Research Support|Eli LIlly: Stocks/Bonds (Public Company)|Gilead: Stocks/Bonds (Public Company)|Sanofi: Grant/Research Support Monika Johnson, BS, AstraZeneca - Icosavax: Grant/Research Support Helen D'Agostino, M.Sc., AstraZeneca - Icosavax: Grant/Research Support Gabriella Alicea, MPH, AstraZeneca - Icosavax: Grant/Research Support Michael Susick, MPH, AstraZeneca - Icosavax: Grant/Research Support Lora Pless, PhD, Astra Zeneca - Icosavax: Grant/Research Support Richard K Zimmerman, MD, MPH, MA, Sanofi: Grant/Research Support GK Balasubramani, PhD, AstraZeneca - Icosavax: Grant/Research Support

